# The role of the Rho/Rock signaling pathway in the pathogenesis of acute ischemic myocardial fibrosis in rat models

**DOI:** 10.3892/etm.2013.935

**Published:** 2013-01-30

**Authors:** HAI-CHENG GAO, HANG ZHAO, WEN-QI ZHANG, YUN-QIAN LI, LI-QUN REN

**Affiliations:** 1 Department of Clinical Pharmacy and Pharmaceutical Management, Jilin University, Changchun 130021;; 2 Department of Neurosurgery, The First Hospital of Jilin University, Changchun 130021;; 3 Department of Cardiology, China-Japan Union Hospital of Jilin University, Changchun 130033, P.R. China

**Keywords:** myocardial fibrosis, Rho, Rho-associated coiled coil-forming protein kinase, isoprenaline hydrochloride

## Abstract

The aim of this study was to investigate the role of the Rho/Rho associated coiled coil-forming protein kinase (Rock) signaling pathway in the pathogenesis of ischemic myocardial fibrosis (MF) in rats. The MF rat model was established using isoprenaline hydrochloride (ISO, 15 mg/kg). Rats were randomly divided into ten groups: a control group and ISO-treated groups at 2 h, 4 h, 6 h, 12 h, 24 h, 48 h, 72 h, 7 days and 21 days. The MF model was evaluated by serum enzyme levels, hematoxylin and eosin (H&E) staining and Masson’s staining, *ex vivo*. The mRNA expression of RhoA and Rock I was assessed with reverse transcription-polymerase chain reaction (RT-PCR). The cell type was evaluated by immunofluorescent and immunohistochemical staining. The protein expression of Rock I was evaluated using western blotting and immunohistochemistry. MF was found to be more developed in the ISO-treated group compared with the control group. CD31 and vimentin expression in fibroblasts and endothelial cells were significantly increased. In addition, the mRNA and protein levels of RhoA and Rock I were significantly increased. In conclusion, activation of Rho/Rock accelerates the degree of ischemic MF. Inhibition of Rho/Rock may be a novel therapeutic strategy for the prevention of ischemic MF.

## Introduction

Myocardial fibrosis (MF) occurs with hypertension, myocardial infarction and heart failure (1). It results from disruption of the equilibrium between synthesis and degradation of collagen and this unbalance leads to an excessive accumulation of collagen fibers within the myocardium (2). MF has an extremely complicated process, which involves inflammatory cytokines and signaling pathways (3).

Rho-associated coiled coil-forming protein kinase (Rock), a member of the serine/threonine kinase family, is the most studied Rho downstream effector molecule. It participates in a variety of cell regulation behaviors and functions, including contraction, transmigration adhesion, growth and splitting, existence, endotheliocyte transformation and fibroblastic hyperplasia (4). A number of abnormal activations of the Rho/Rock pathway are present in diseases (5). The Rho/Rock pathway is activated by multiple cytokines and inflammatory mediators, including platelet-derived growth factor, transforming growth factor-*β*, endothelin-1 and angiotensin II (6). These cytokines and vascular active materials mediate a number of inflammatory lesions and fibre hyperplasia diseases. Therefore, the Rho/Rock pathway may be involved in the after effects of inflammatory mediators and therefore may participate in the pathophysiological process of these diseases.

Previous studies have suggested that the Rho/Rock pathway also plays a significant role in bowel fibrosis, liver fibrosis and renal fibrosis. Hudson (7) reported that in the development process of bowel fibrosis, the function of connective tissue growth factor (CTGF) was associated with the activation of Smads, Rho/Rock, mitogen-activated protein kinase (MAPK) and protein kinase C (PKC). Murata *et al* (8) reported that the specific Rock blocker Y-27632 prevents the activation of hepatic stellate cells (HSC) and rat liver fibrosis. Tian and Kaufman (9) identified that Rho exists in the cytoplasm in the non-active form combined with guanosine diphosphate (GDP); however, it has an effect on intracellular effective factors in the active form combined with guanosine triphosphate (GTP). Nagatoya *et al* (10) identified that the development of renal tubular interstitial fibrosis is prevented by blocking the Rho/Rock pathway. However, the function of the Rho/Rock pathway in ischemic MF remains unclear.

The present study focused on the expression and function of the Rho/Rock pathway in ischemic MF of rats. Additionally, the pathogenesis of MF was explored, in order to provide valuable data for clinical practice.

## Materials and methods

### Materials

Streptomycin avidin peroxidase immunohistochemistry (SP-IHC) kits of RhoA, Rock I, vimentin and CD31 were purchased from Zhongshan Biotechnology Co., Ltd. (Beijing, China). All other chemicals used were of analytical grade from commercial suppliers in China.

### Animals and MF modeling

**Figure 1:**
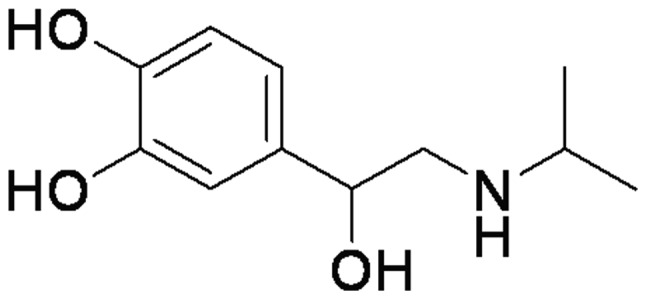
Molecular structure of isoprenaline hydrochloride (ISO).

Fifty male Wistar rats (SCXKJ2007-0003; 180–220 g) were purchased from Jilin University Laboratory Animal Center and were randomized into ten groups: control and model groups at 2 h, 12 h, 7 days and 21 days (n=5, respectively). The MF rat model was established by peritoneal injection of ISO (Fig. 1) on 25th June 2011 in the College of Pharmacy, Jilin University and the control group was injected with the same volume of 0.9% NaCl. MF and control rats were sacrificed at 2 h, 12 h, 7 days and 21 days, respectively. At the end of the experiments, rats were sacrificed and their hearts were removed. A portion of the heart was fixed in 10% phosphate-buffered formalin for histological studies. Another portion was snap-frozen in liquid nitrogen and stored at −80°C for extractions. The study was approved by the ethics committee of the institution.

### Analysis of serum enzymes

Serum aspartate aminotransferase (AST), lactic dehydrogenase (LDH), creatine kinase (CK) and creatine kinase isozyme (CK-MB) activities were measured using the MD-100 Multifunctional Automatic Biochemistry Analyzer (Sanhe Medical Equipment Co., Ltd., Dandong, China) according to the manufacturer’s instructions.

### Hematoxylin and eosin (H&E) and Masson’s staining

Renal histology was assessed by light microscopy with H&E staining and Masson’s trichrome staining. Ten high-power microscopic fields were randomly selected. Fibrosis was quantified and compared between the MF and control groups.

### Reverse transcription-polymerase chain reaction (RT-PCR)

Total RNA was extracted from the heart tissues. Primers for RhoA, Rock I and glyceraldehyde 3-phosphate dehydrogenase (GAPDH) were designed and synthesized by Shanghai Sangon Biological Engineering Technology and Services Co., Ltd., (Shanghai, China). The sequences for these primers are presented in Table I. Total RNA (0.5 *μ*g) was amplified using the Titan™ One Tube RT-PCR kit (Boehringer-Mannheim, Shanghai, China). The amplification consisted of 30 cycles. The products were separated by agarose gel electrophoresis and visualized by ethidium bromide staining. Bands were digitized using a Tanon-1000 Gel Image System (Shanghai, China). The ratios of RhoA and Rock I band grayscales to GAPDH band grayscales in the various groups were measured.

### Western blotting

Western blotting was performed as previously described (11). Briefly, heart tissues were homogenized in protein lysis buffer and 50 g proteins were separated on 10% sodium dodecyl sulfate (SDS) gels and electroblotted to polyvinylidine fluoride membranes. The membranes were blocked with skimmed milk powder solution for 2 h. Blots were incubated using goat anti-mouse anti-Rock I monoclonal antibody (1:400 dilution) at 4°C overnight, followed by peroxidase-conjugated rabbit anti-goat antibody. Color was developed with enhanced chemiluminescence (ECL) in a dark room. The grayscales were analysed using the Tanon-1000 Gel Image System.

### Immunofluorescent and immunohistochemical staining

The paraffin-embedded tissue sections (0.2 *μ*m) were deparaffinized with xylene and rehydrated with graded washes of ethanol to phosphate-buffered saline (PBS). Then, each slice was treated with 30 *μ*l 3% H*_2_*O*_2_* (reagent A), incubated at room temperature for 20 min and washed twice with PBS. Then, 30 *μ*l goat serum (reagent B) was added, followed by incubation at room temperature for 20 min and two washes with PBS. Each slice was incubated in 30 *μ*l primary antibody (mouse anti-rat Rock I monoclonal antibody, 1:200 dilution) and placed in the wet box at 4°C overnight. After washing with PBS, the slices were incubated in 30 *μ*l biotinylated polyclonal secondary antibody (reagent C) at room temperature for 30 min, followed by washing with PBS. The diaminobenzidine (DAB) method was used for color development, followed by washing with tap water. Slices were restained with hematoxylin, incubated in ammonia, dehydrated with gradient ethanol, transparentized with xylene and finally sealed with neutral gum. The cells with brown particles in their cytoplasm and nucleus were denoted positive under a light microscope.

### Statistical analysis

All experiments were performed at least three times. Data were presented as mean ± standard error of the mean (SEM). All statistical analyses were performed using SPSS 11.5 for Windows (SPSS Inc., Chicago, IL, USA). Comparisons between multiple groups were performed by one-way analysis of variance (ANOVA). P<0.05 was considered to indicate a statistically significant difference.

## Results

### Diagnostic serum enzymes

Two hours after ISO injection, the activity of serum AST, LDH, CK and CK-MB increased when compared with the control group; however, only changes in AST and CK-MB expression were statistically significant (P<0.01, P<0.05, respectively). Four hours after ISO injection, AST continuously increased, LDH, CK and CK-MB expression peaked at 24 h after ISO injection, and LDH, CK and CK-MB became less active. For three weeks, the activities of these substances became steady and remained significantly higher than the control. AST reached the peak 6 h after ISO injection (P<0.01) and gradually decreased 12 h later, still significantly higher than the control. These results demonstrate that ISO (15 mg/kg) successfully causes myocardial ischemia in rats (Table II).

### Light microscope examination

**Figure 2:**
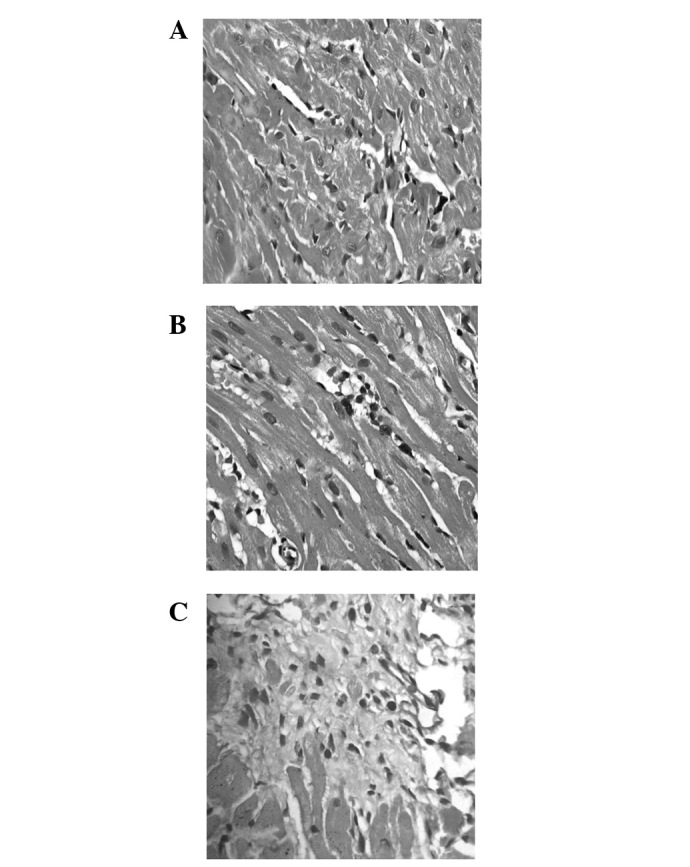
H&E staining of myocardial tissue in rats (magnification, ×400). (A) Control group presenting normal cardiac fibres without any ischemia or MF; (B) 2 h after ISO injection; (C) 21 days after ISO injection. H&E, hematoxylin and eosin; ISO, isoprenaline hydrochloride.

A morphological assessment, as the golden standard for disease diagnosis, is the most reliable method for diagnosing MF and its development (12). Examination of the rat heart tissue slices revealed that that there was clear denaturation and dropsy in the apex cordis and endocardium. Sporadic spotty necrosis was observed in the endocardium 2 h after ISO injection. Four hours after ISO injection, increased denaturation, appearance of small necrotic foci and broken myofibril structure were observed at the apex cordis, endocardium and around blood vessels. Additionally, there was phagocyte infiltration and coagulative myolysis. Forty-eight hours after ISO injection, widespread, multiple and sporadic necrotic foci with clear boundaries were observed. Fibroblasts were rich in these foci and the extent of necrosis became gradually aggravated. One week after ISO injection, multiple and sporadic necrotic foci with clear boundaries were observed around coronary artery branching. In the foci, there was cellular infiltration in monocytes, lymphocytes and neutrophils; the fibroblasts proliferated and a certain amount of collagen fiber was observed. At three weeks, the level of necrosis peaked and a large amount of fibrosis was observed. This indicates that ISO induced MF in rats, as shown in Fig. 2, including prominent interstitial expansion, inflammatory cell infiltration and interstitial collagen accumulation (12 h-21 days). In addition, the myocardium of rats presented a significant degree of fibrosis at 21 days (Fig. 2C). These results show that we succeeded in creating an MF rat model.

### Masson’s trichrome staining

**Figure 3:**
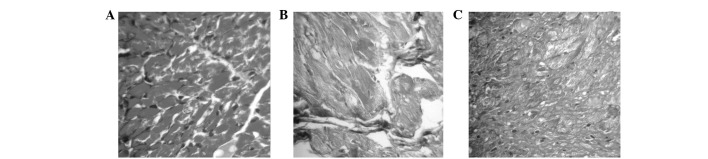
Masson’s trichrome staining of myocardial tissue in rats (magnification, ×400). (A) Control group; (B) 2 h after ISO injection; (C) 21 days after ISO injection. ISO, isoprenaline hydrochloride.

The pathological features with Masson’s trichrome staining of the ISO-treated groups included girdle-shaped collagen in the interstitium of the heart, particularly on day 12 in the ISO-treated rats (Fig. 3A–C). Masson’s staining presented collagen fibers as green, muscle fibers as red and red blood cells as jacinth. Green collagen fibers were mainly observed at the apex of the heart, subendocardial necrosis foci and around larger vessels among the muscle bundles. In addition, only a small portion of collagen expression was identified at the myocardial tissue of the control group. Two hours after ISO injection, the number of collagen fibers was greater than the control group. For three weeks, the number of collagen fibers significantly increased. These results further confirm the results presented in serology and H&E staining (Fig. 3)

### Expression of RhoA and Rock I

**Figure 4:**
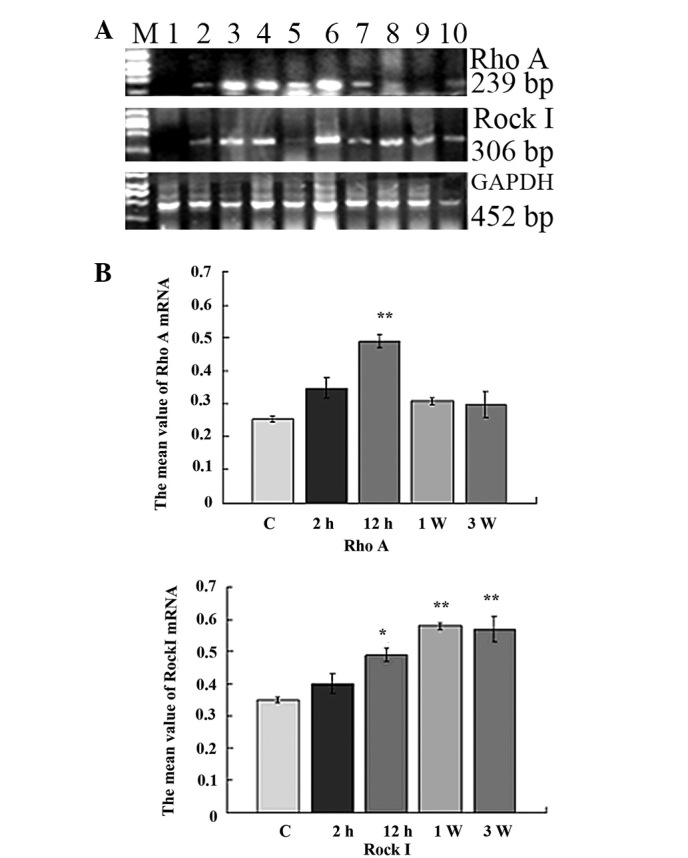
Expression of RhoA and Rock I in myocardial tissue detected by RT-PCR. (A) M, DNA marker 2000; lane 1, Control group; lanes 2–10, 2 h, 4 h, 6 h, 12 h, 24 h, 48 h, 72 h, 7 days and 21 days after ISO injection. (B) Grayscale values. ^*^P<0.05, ^**^P<0.01 vs. the control group. Rock, Rho-associated coiled coil-forming protein kinase; RT-PCR, reverse transcription-polymerase chain reaction; ISO, isoprenaline hydrochloride; GAPDH, glyceraldehyde 3-phosphate dehydrogenase.

**Figure 5:**
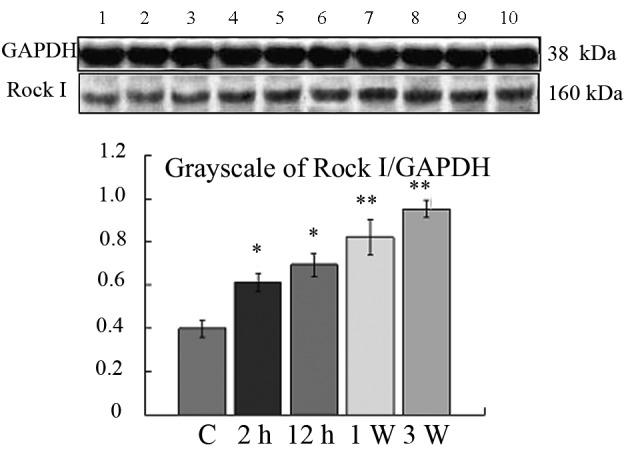
Western blotting of Rock I. Lane 1, control group; lanes 2–9, 2 h, 4 h, 6 h, 12 h, 24 h, 48 h, 72 h, 7 days and 21 days1 days after ISO injection. ^*^P<0.05, ^**^P<0.01 vs. the control group. Rock, Rho-associated coiled coil-forming protein kinase; ISO, isoprenaline hydrochloride; GAPDH, glyceraldehyde 3-phosphate dehydrogenase.

Semi-quantitative RT-PCR analysis was performed on the heart tissues. The products of RhoA and Rock I are presented in Fig. 4B. The bands were analyzed by densitometry and the target transcript levels were normalized against GAPDH (Fig. 4A). Two hours after ISO injection, the expression of RhoA and Rock I in the myocardium had gradually increased compared to that in the control group. No significant difference was identified between the control and ISO-treated groups. Twelve hours after ISO injection, RhoA and Rock I mRNA expression had markedly increased (Fig. 5; P<0.01 and P<0.05, respectively). The mRNA expression of RhoA reached the maximum value 12 h after ISO injection (P<0.01). The mRNA expression of Rock I reached a maximum value at 7 days (P<0.01). These results indicate that the Rho/Rock pathway plays an important role in MF formation (Fig. 4).

Western blotting was used to investigate the expression of Rock I. The results were analyzed semi-quantitatively according to the grayscale value. As shown in Fig. 5, the expression level of Rock I in the control group was undetectable in SDS-polyacrylamide gel electrophoresis (PAGE). The expression of Rock I in the ISO-treated group from 2 h to 21 days increased significantly when compared to the control group (Fig. 5; P<0.05 and P<0.01, respectively). These results validate the mRNA expression of Rock I.

### Immunofluorescence of CD31 expression

**Figure 6:**
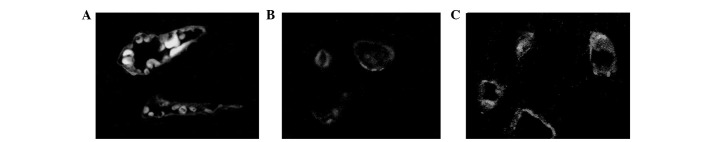
CD31 staining of myocardial tissue in rats. (magnification, ×400). (A) Control group; (B) 2 h after ISO injection; (C) 21 days after ISO injection. ISO, isoprenaline hydrochloride.

As shown in Fig. 6, CD31 was highly specific and sensitive for vascular endotheliocytes. Fibrosis triggered the CD31 expression of endotheliocytes.

### Expression of vimentin

**Figure 7:**
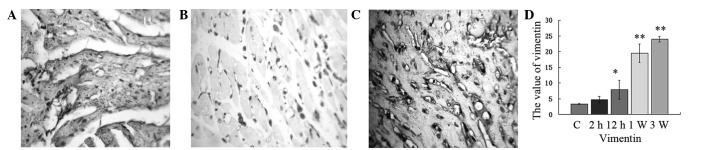
Immunohistochemical assay of vimentin in myocardial tissue of rats (magnification, ×400). (A) Control group; (B) 2 h after ISO injection; (C) 21 days after ISO injection; (D) grayscale values. ^*^P<0.05, ^**^P<0.01 vs. the control group. ISO, isoprenaline hydrochloride.

We examined the change in vimentin protein expression following ISO injection. Staining revealed that the cytoplasm around the nucleus was brown and granular and the nucleus was not stained. A few vimentin-positive cells were observed among the myocardial myofibrils of the control group. Two hours after ISO injection, the fraction of vimentin-positive cells did not increase. From 12 h to 21 days after ISO injection, a large number of vimentin-positive cells were observed in necrosis foci, which increased gradually. The expression of vimentin significantly increased from 2 h to 21 days and reached the maximum when compared with the control group at 21 days (Fig. 7; P<0.01). In addition, the cells became fibroblasts. These results indicate that fibroblasts play an important role in MF.

### Expression of Rock I

**Figure 8:**
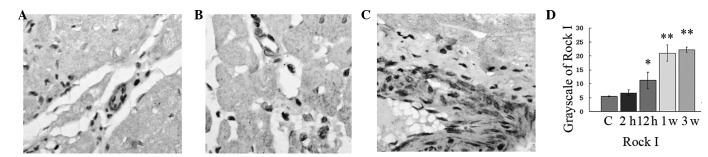
Immunohistochemical assay to assess Rock I expression. (A) Control group; (B) 2 h after ISO injection; (C) 21 days after ISO injection; (D) grayscale values. ^*^P<0.05, ^**^P<0.01 vs. the control group. Rock, Rho-associated coiled coil-forming protein kinase; ISO, isoprenaline hydrochloride.

Histopathological examination of the myocardium of the control group revealed clear integrity of the myocardial cell membrane and normal cardiac fibers without any infarction or fibrosis. No inflammatory cell infiltration was observed (Fig. 8). In the ISO-treated group, widespread loss of myofibers, focal myonecrosis and marked edema with moderate infiltration of lymphocytes and macrophages were observed (P<0.01; Fig. 8). The protein expression of Rock I reached the maximum value at 21 days (P<0.01). In addition, the expressional cell type was endotheliocytes and fibroblasts (Fig. 8). Rock I played an important role in MF formation. Simultaneously, the expression of CD31 and vimentin validated the expression of Rock I.

## Discussion

The pathogenesis of acute MF is not fully understood. Studies on ISO-induced MF provide a good insight into the pathology of MF and clearly indicate the involvement of oxidative stress (13). The myocardium contains various enzyme systems. When the myocardium is traumatized, enzymes are released into the blood and increase the activities of these enzymes in the serum. These enzymes are called myocardial enzymes (14). The combination of several enzymes that are closely related to the myocardium is called myocardial enzyme spectrum. Although none of the enzyme spectrum is specific to the myocardium, they have specificity for the diagnosis of myocardium trauma (15). Clinically, the level of cardiac enzymes is used indirectly to measure the trauma of cardiocytes. In the present study, serum enzymes and H&E staining in myocardial tissue in the ISO-treated group were significantly higher than in the control group. Excessive collagen accumulation in the myocardium was observed in the ISO-treated group. These findings demonstrate that the MF rat model was successfully induced by injection of 15 mg/kg ISO and this model mimics ischemic MF in humans.

CD31 is a 130 kDa integral membrane protein, a member of the immunoglobulin superfamily that mediates cell-to-cell adhesion (16). CD31 is expressed constitutively on the surface of adult and embryonic endothelial cells and is weakly expressed on numerous peripheral leukocytes and platelets. In the present study, when there is MF, CD31 was expressed in endothelial cells.

Vimentin is expressed in a wide variety of mesenchymal cell types, including fibroblasts and endothelial cells (16). Rho/Rock controls a wide variety of signal transduction pathways (17). Rock exists as two isoforms, Rock I and Rock II. They share an overall homology of 92% in their kinase domains. Two major families of Rock inhibitors, fasudil and Y-27632, are extensively used. Rock not only regulates the actin cytoskeleton, but also the expression of genes associated with tissue fibrosis (18). Data from one study suggests that diabetes impairs cardiac function through upregulation of RhoA (19). The increase in RhoA expression and activity in diabetic hearts results in increased phosphorylation of Rock targets (19). In the present study, the mRNA and protein expression of RhoA and Rock I were significantly increased. The biological effector cells of RhoA and Rock I were assessed by specific staining. The results revealed a large number of active fibroblasts and endotheliocytes around the deposits in MF rats. These findings show that the Rho/Rock signaling pathway plays an important role in MF formation. Inhibition of Rho/Rock may be a novel therapeutic target for prevention of ischemic MF. Further research is required to clarify the exact molecular signaling pathways.

## Figures and Tables

**Table I:** Upstream and downstream primer sequences for GAPDH, RhoA and Rock I.

Primer	Sequence	Length (bp)	Temperature (°C)
GAPDH	5′-ACCACAGTCCATGCCATCAC-3′		
	5′-TCCACCACCCTGTTGCTGTA-3′	452	55
RhoA	5′-GATGGAGCTTGTGGTAAGA-3′		
	5′-AAACTATCAGGGCTGTCG-3′	239	55
Rock I	5′-GCACACTGGCAATGTAATGC-3′		
	5′-GTTGAACAGAACAAGTGACC-3′	302	55

GAPDH, glyceraldehyde 3-phosphate dehydrogenase; Rock, Rho-associated coiled coil-forming protein kinase.

**Table II:** Diagnostic serum enzymes (U/l).

Group	AST	LDH	CK	CK-MB
Control	83.4±7.8	251±90.4	588.2±228.3	575.1±118.6
ISO				
2 h	125.0±14.8*^a^*	271.2±53.6	646.2±116.9	761.5±64.4^b^
4 h	167.8±37.6*^a^*	586±323.2*^a^*	1283.2±258.1*^a^*	762.9±62.6^b^
6 h	183±27.6*^a^*	368.2±128.6	581.0±222.6	745.6±130.0 ^b^
12 h	144.4±32.4*^a^*	425.6±140.0*^a^*	575.0±134.3	672.4±105.9
24 h	96.4±8.1	305.2±71.0	528.6±131.9	652.7±78.5
48 h	123.4±39.0*^a^*	391.4±147.4	549.8±248.6	469.4±109.0
72 h	96.2±6.4	466.0±87.0*^a^*	722.2±95.6	727.4±202.0^b^
7 days	100.0±10.4	327.4±73.1	633.2±134.1	727.6±10.0
21 days	100.6±9.7	335.6±90.7	644±211.9	644.1±99.6

a P<0.01,

b P<0.05 compared to the control. Data are presented as mean ± standard deviation. AST, aspartate amino transferase; LDH, lactic dehydrogenase; CK, creatine kinase; CK-MB, creatine kinase isozyme; ISO, isoprenaline hydochloride.
